# Robust tests for combining *p*-values under arbitrary dependency structures

**DOI:** 10.1038/s41598-022-07094-7

**Published:** 2022-02-24

**Authors:** Zhongxue Chen

**Affiliations:** Department of Epidemiology and Biostatistics, School of Public Health, Indiana University Bloomington, 1025 E. 7th Street, Bloomington, IN 47405 USA

**Keywords:** Software, Statistics

## Abstract

Recently Liu and Xie proposed a *p*-value combination test based on the Cauchy distribution (CCT). They showed that when the significance levels are small, CCT can control type I error rate and the resulting *p*-value can be simply approximated using a Cauchy distribution. One very special and attractive property of CCT is that it is applicable to situations where the *p*-values to be combined are dependent. However, in this paper, we show that under some conditions the commonly used MinP test is much more powerful than CCT. In addition, under some other situations, CCT is powerless at all. Therefore, we should use CCT with caution. We also proposed new robust *p*-value combination tests using a second MinP/CCT to combine the dependent *p*-values obtained from CCT and MinP applied to the original *p*-values. We call the new tests MinP-CCT-MinP (MCM) and CCT-MinP-CCT (CMC). We study the performance of the new tests by comparing them with CCT and MinP using comprehensive simulation study. Our study shows that the proposed tests, MCM and CMC, are robust and powerful under many conditions, and can be considered as alternatives of CCT or MinP.

## Introduction

*P*-value combination approaches are important and critical in statistical inference, especially in statistical hypothesis testing^1–5^. Many commonly used tests are special cases of *p*-value combination methods. For example, a chi-square test statistic with $k (k>1)$ degrees of freedom (df) under the null hypothesis can be decomposed as $k$ components each has identical and independent chi-square distribution with 1 df^6,7^. Therefore, we can get $k$ *p*-values each from the individual component. Then the original chi-square test with k df can be viewed as being obtained through the Lancaster’s generalized Fisher chi-square test which obtains the $k$ components from the $k$ *p*-values and their sum is identical to the original test statistic^5,8^. Most recently, a class of *p*-value combination tests based on gamma distribution have been proposed and studied^9^. This class of tests includes some existing popular tests, such as the MinP test, Fisher test, and z test, as special cases. With recent advancements in biotechnologies, huge amount of data has been generated in genetics and genomics studies. For instance, in genome-wide association studies (GWAS) to identify genetic risk factors associated with given disease, many gene- or set-based association tests have been developed which utilize the approaches for combining *p*-values^10–14^. How to combine these *p*-values is still a challenging topic in the area. Therefore, developing appropriate powerful and robust *p*-value combination tests is extremely important in statistical practice. Although the commonly used MinP test is robust and applicable to independent and dependent *p*-values^1^, under some conditions, it might be less powerful than other tests^9^.

Recently, a *p*-value combination test based on Cauchy distribution, CCT, was proposed^15^ and immediately attracted a lot of attentions. This test has been applied in many areas^16–19^. The authors claimed, as shown in the title, that CCT is powerful under arbitrary dependency structures of the *p*-values. They also proved that when the significance levels are small, CCT can control type I error rate and its *p*-value can be easily calculated analytically based on a Cauchy distribution. However, under some conditions, for instance, when one-sided *p*-values are obtained and some of them are very large due to the wrong direction assumed, CCT may be less powerful than the MinP test. In this paper, we further study this test and its properties. Furthermore, we propose two robust and powerful tests as alternatives of CCT to combine dependent *p*-values. The paper is organized as follows. In the second section, we present CCT and the proposed tests with some details. In section “Results”, we compare the performances of those tests using a comprehensive simulation study and real data application. Some discussions and conclusion are given in the last section.

## Method

Assume we want to combine $k$ *p*-values,${P}_{1},{ P}_{2}, \ldots , { P}_{k}$, through testing the global null hypothesis ${H}_{0}={\bigcap }_{i}{H}_{i,0}$ against the global alternative hypothesis ${H}_{1}={\bigcup }_{i}{H}_{i,1}$, where ${H}_{i,0}$ and ${H}_{i,1}$ are the individual null and alternative hypotheses, respectively, for study $i (i={1,2},\ldots ,k)$. We also assume that under ${H}_{0}$, each ${P}_{i}\sim U({0,1})$, a uniform distribution between 0 and 1. Using the standard Cauchy distribution, C(0,1), we can first transform the *p*-values to ${T}_{i}=\tan[(0.5-{P}_{i})\pi ]$ for $i={1,2},\ldots ,k$. Then under the global null hypothesis, ${T}_{i}\sim C({0,1})$. Denote the ordered *p*-values, ${P}_{(1)}\le {P}_{(2)}\le \cdots \le {P}_{(k)}$, and their corresponding transformed values from the Cauchy distribution as ${T}_{(1)}, {T}_{(2)},\ldots ,{T}_{(k)}$. We have ${T}_{(1)}\ge {T}_{(2)}\ge \cdots \ge {T}_{(k)}$.

The CCT is constructed using the following test statistic^15^: $T=\sum_{i=1}^{k}{{w}_{i}T}_{i}$, where ${w}_{i}\ge 0$ are the weights satisfying $\sum_{i=1}^{k}{w}_{i}=1$. And the *p*-value from the CCT is calculated as ${p}_{CCT}=P[C\left({0,1}\right)\ge t]$, where $t=\sum_{i=1}^{k}{{w}_{i}t}_{i}$ is the observed test statistic of $T$. For the CCT, we have the following new results.

### Theorem 1

$${P}_{(1)}\le {P}_{CCT}\le {P}_{(k)}$$.

### Proof

$T=\sum_{i=1}^{k}{{w}_{i}T}_{i}\le \sum_{i=1}^{k}{w}_{i}{T}_{\left(1\right)}={T}_{\left(1\right)}$, and $T=\sum_{i=1}^{k}{{w}_{i}T}_{i}\ge \sum_{i=1}^{k}{w}_{i}{T}_{\left(k\right)}={T}_{\left(k\right)}$. Therefore, ${P}_{CCT}=P\left[C\left({0,1}\right)\ge T\right]\ge P\left[C\left({0,1}\right)\ge {T}_{\left(1\right)}\right]={P}_{(1)}$, and ${P}_{CCT}=P\left[C\left({0,1}\right)\ge T\right]\le P\left[C\left({0,1}\right)\ge {T}_{\left(k\right)}\right]={P}_{(k)}$.

### Remark 1

Theorem 1 implies that the CCT test can’t provide stronger evidence (i.e., smaller *p*-value) to reject the global null hypothesis than the strongest one that against an individual null hypothesis.

### Remark 2

Because of the fact stated in Remark 1, CCT is not preferable for combining independent *p*-values.

### Theorem 2

*At small significance level, CCT can control type I error rate for p-values under arbitrary dependency structures, i.e.,* $\underset{t\to \infty }{\mathrm{lim}}\frac{P\left[T\ge t\right]}{P\left[C({0,1})\ge t\right]}\le 1$.

### Proof

$$P\left[T<t\right]=P\left[\sum_{i=1}^{k}{{w}_{i}T}_{i}<t\right]\ge P\left[{T}_{\left(1\right)}<t\right]=1-P\left[{T}_{\left(1\right)}\ge t\right]=1-P\left[\bigcup_{i=1}^{k}({T}_{i}\ge t)\right]\ge 1-\sum_{i=1}^{k}P\left[{T}_{i}\ge t\right]=1-k\left(1-P\left[C\left({0,1}\right)<t\right]\right)=1-k+kP\left[C\left({0,1}\right)<t\right]$$,

Hence, $\lim\nolimits_{t\to \infty} \frac{P\left[T<t\right]}{P\left[C\left({0,1}\right)<t\right]}\ge \lim\nolimits_{t\to \infty} \frac{1-k+kP\left[C\left({0,1}\right)<t\right]}{P\left[C\left({0,1}\right)<t\right]}=1$, or $\lim\nolimits_{t\to \infty} (P\left[T<t\right]-P\left[C\left({0,1}\right)<t\right])\ge 0$, $\lim\nolimits_{t\to \infty} (P\left[T\ge t\right]-P\left[C\left({0,1}\right)\ge t\right])\le 0$, and $\lim\nolimits_{t\to \infty }\frac{P\left[T\ge t\right]}{P\left[C({0,1})\ge t\right]}\le 1$.

### Remark 3

The same result as in Theorem 2 was also proved in other papers^15,16^, but they made some distributional assumptions about the $T_{i}^{\prime } s$. Here we provide a new proof without any additional assumptions (i.e., under truly arbitrary dependency structures of the *p*-values to be combined).

### Remark 4

Theorem 2 proves that CCT can control type I error rate at small significance level for arbitrary dependency structures of the *p*-values to be combined. However, it may not be powerful, or even powerless, under some conditions. For instance, if ${p}_{1}+{p}_{2}=1$ (e.g., ${p}_{1} \text{ and } {p}_{2}$ are *p*-values from left- and right-sided t-test), then the test statistic from the CCT will be 0 and its *p*-value ${p}_{CCT}=P\left[C\left({0,1}\right)\ge 0\right]=0.5$. Therefore, for any significance level less than 0.5, the power value will be 0 (i.e., the type II error rate will be 1). Interestingly, under this condition, the MinP test gives the same *p*-value obtained from the two-sided test. This simple example also indicates that the main result, Theorem 1 of Liu and Xie^15^, may not be valid any longer if the assumptions made in their paper are violated. In other words, CCT is not always powerful to combine *p*-values under arbitrary dependency structures.

From the construction of the test statistic, we see that CCT may gain some power if all the *p*-values to be combined are small and/or positively correlated. However, as mentioned above CCT may be much less powerful than the MinP test, which is known for its robustness but conservative in general. To incorporate the good properties from both CCT and MinP, we propose the following two tests. The first one is called MinP-CCT-MinP (MCM), whose *p*-value is calculated as: ${p}_{MCM}=2\min\{{p}_{CCT},{p}_{MinP}, 0.5\}$, where ${p}_{MinP}$ is obtained by applying MinP to the original *p*-values to be combined. The second one is called CCT-MinP-CCT-(CMC), whose *p*-value is calculated as: ${p}_{CMC}=CCT\{{p}_{CCT}, {p}_{MinP}\}$.

Since both CCT and MinP can control type I error for small significance level, and MCM and CMC are the MinP and CCT to combine their *p*-values, respectively, we have the following result.

### Theorem 3

*For small significance level, both MCM and CMC can control type I error rate for p-values under arbitrary dependency structures.*

## Results

To study the performances of MCM and CMC, we conduct a comprehensive simulation study by comparing these tests with CCT and MinP. We also apply the new tests to some real data to demonstrate their usefulness.

### Simulation study

In the simulation study, following the settings in Liu and Xie^15^, we assume the random vector ${X}^{T}=({X}_{1},{X}_{2},\ldots ,{X}_{k})$ has a multivariate normal distribution with correlation matrix $\Sigma =({\sigma }_{ij})$. For the correlation matrix, we consider three different models.

**Model 1** (AR(1) correlation, “Expo”): ${\sigma }_{ij}={\rho }^{|i-j|}$ for $1\le i,j\le k$, where $\rho$ is a constant between 0 and 1.

**Model 2** (polynomial decay, “Poly”): ${\sigma }_{ii}=1$ and ${\sigma }_{ij}=\frac{1}{0.7+{|i-j|}^{r}}$ for $1\le i\ne j\le k$.

**Model 3** (Singular matrix, “SiG”): Let $A=({a}_{ij})$ be a $k/5\times k$ matrix where ${a}_{ij}={d}^{|i-j|}$ and $d$ is a constant between 0 and 1. Let $D=({d}_{ij})$ be a diagonal matrix with diagonal elements ${d}_{ii}={({\tilde{a }}_{ii})}^{-1/2}$, where ${\tilde{a }}_{ii}$ is the ${i}{th}$ diagonal of ${A}^{T}A$. The correlation matrix is then $\Sigma ={D}^{T}{A}^{T}AD$.

For the above three models of the correlation matrix $\Sigma$, we use different values for the parameters ($\rho , r,\text{ and } d$). We also choose different numbers of *p*-values (i.e., $k$) in the simulation study. To investigate how the tests control type I error rate, we simulate $X\sim MVN(0,\Sigma )$ with $\Sigma$ being in one of the three above models. For the power comparison, under the global alternative hypothesis ${H}_{1}$, we assume a subset of the vector $X$ has non-zero mean. Of those significant random variables, we also assume some of them have negative mean ($-\mu$) and the rest have positive mean ($\mu$). For each variable ${X}_{i}$ three different *p*-values, according to three types of individual alternatives (${\mu }_{i}<0, {\mu }_{i}>0,\; {\text{and}}\; {\mu }_{i}\ne 0,$ respectively), are calculated: left-sided *p*-value $\Phi ({X}_{i})$, right-sided *p*-value $1-\Phi ({X}_{i})$, and two-sided *p*-value $2\Phi \left({-|X}_{i}|\right),$ where $\Phi (\cdot )$ is the cumulative distribution function of the standard normal distribution. All the tests are then applied to the three sets of *p*-values.

Table 1 displays the empirical type I error rate (divided by the significance level) for all of the tests applied to the left-sided *p*-values using different significance levels. All of the tests control type I error rate, except for CCT which may have slightly higher type I error rates when the preset significance levels are large, this pattern was also observed by Liu and Xie^15^. It is also noticeable that under some conditions, MinP, MCM and CMC may have lower type I error rates than expected. Similar patterns are observed when these tests are applied to the right-sided and two-sided *p*-values (data not shown). The similar patterns are also observed under other simulation settings (see Tables S1–S2 in supplementary materials).

**Table 1:** Empirical type I error rate (/significance level) using 1e^6^ replicates, highlighted bold are the values greater than the significance level and outside of the 95% CI.

Model	Number of *p*-values ($k$)	Test	Significance level			
			0.05	0.01	0.001	0.0001
Expo ($\rho$=0.5)	5	CCT	**1.16**	**1.10**	**1.08**	1.07
		MinP	0.88	0.93	1.00	1.05
		MCM	0.58	0.54	0.52	0.60
		CMC	1.01	1.01	1.04	1.06
	10	CCT	**1.18**	**1.11**	**1.09**	1.03
		MinP	0.88	0.94	1.01	1.00
		MCM	0.58	0.55	0.52	0.54
		CMC	1.01	1.02	1.04	1.03
	20	CCT	**1.18**	**1.09**	1.00	1.04
		MinP	0.90	0.94	0.95	1.02
		MCM	0.58	0.54	0.47	0.46
		CMC	1.01	1.00	0.97	1.04
	100	CCT	**1.11**	**1.05**	1.04	1.06
		MinP	0.93	0.97	1.02	1.05
		MCM	0.56	0.53	0.54	0.42
		CMC	1.00	1.00	1.03	1.04
Poly ($r=1.5$)	5	CCT	**1.16**	**1.12**	**1.07**	1.10
		MinP	0.83	0.90	0.98	1.03
		MCM	0.58	0.55	0.52	0.69
		CMC	0.99	1.00	1.02	1.04
	10	CCT	1.21	1.13	1.04	0.84
		MinP	0.84	0.90	0.94	0.79
		MCM	0.60	0.55	0.51	0.40
		CMC	1.01	1.01	1.00	0.80
	20	CCT	1.23	1.13	1.02	0.95
		MinP	0.85	0.92	0.93	0.89
		MCM	0.60	0.54	0.52	0.43
		CMC	1.02	1.01	0.97	0.94
	100	CCT	1.17	1.05	0.98	0.99
		MinP	0.89	0.92	0.92	0.97
		MCM	0.57	0.52	0.47	0.44
		CMC	1.00	0.98	0.94	0.99
SiG ($d=0.5$)	5	CCT	0.60	0.60	0.61	0.53
		MinP	0.40	0.40	0.37	0.24
		MCM	0.40	0.40	0.38	0.29
		CMC	0.40	0.40	0.41	0.31
	10	CCT	0.76	0.82	0.86	0.84
		MinP	0.34	0.36	0.38	0.39
		MCM	0.45	0.47	0.50	0.52
		CMC	0.51	0.56	0.61	0.62
	20	CCT	0.87	0.93	1.04	1.05
		MinP	0.29	0.30	0.32	0.38
		MCM	0.48	0.49	0.53	0.60
		CMC	0.56	0.60	0.68	0.69
	100	CCT	0.98	1.00	1.01	0.99
		MinP	0.22	0.23	0.22	0.27
		MCM	0.50	0.51	0.50	0.43
		CMC	0.49	0.62	0.62	0.59

**Figure 1:**
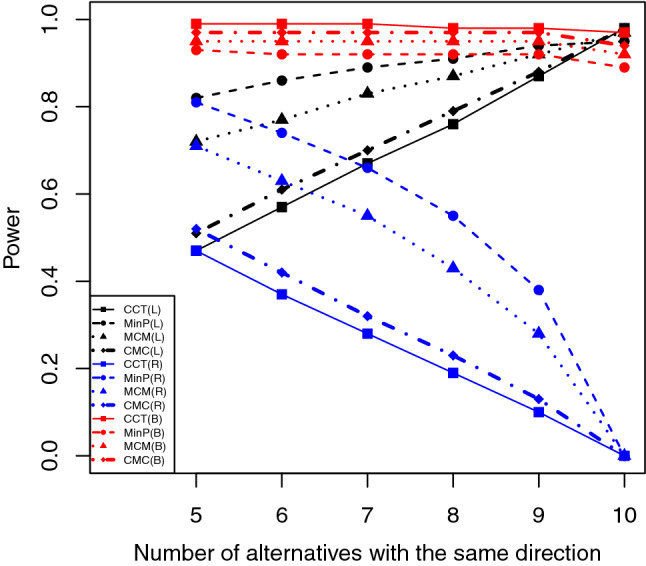
Empirical power when there are 10 out of 20 *p*-values are significant with model = “Expo”, $\rho =0.5$, and $\mu =2$.

**Figure 2:**
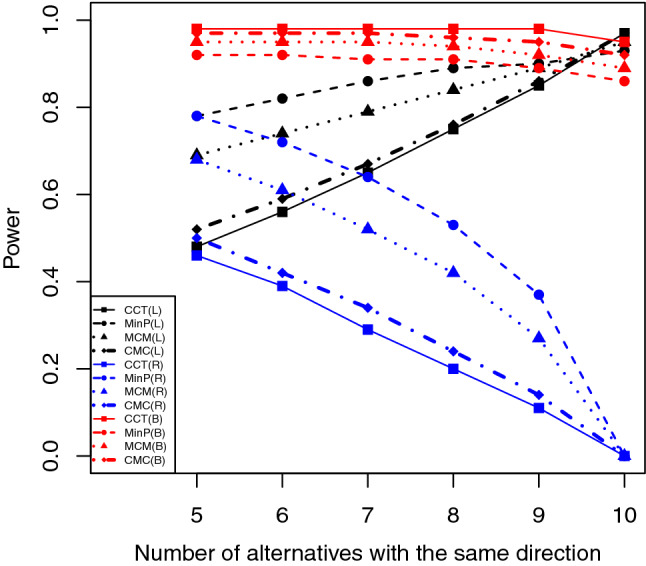
Empirical power when there are 10 out of 20 *p*-values are significant with model = “Poly”, $r=1.5$, and $\mu =2$.

**Figure 3:**
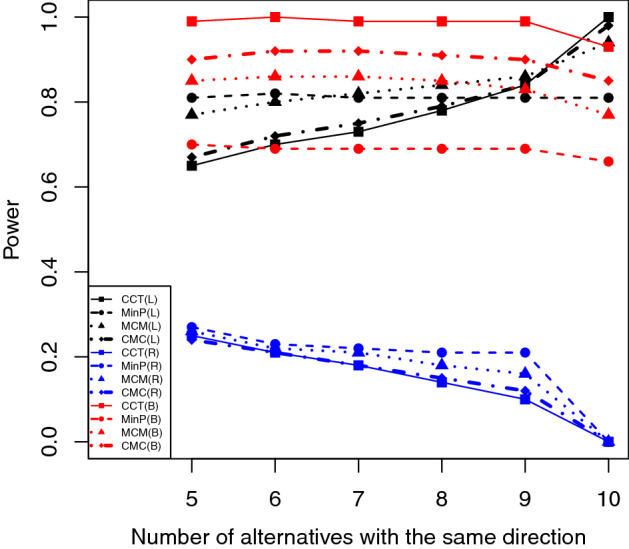
Empirical power when there are 10 out of 20 *p*-values are significant with model = “SiG”, $d=0.5$, and $\mu =2$.

Figures 1, 2 and 3 show the empirical powers for each test under different conditions when the significance level of 0.05 was used. We observe the following patterns. First, when one-sided (left- and right-sided) *p*-values are used, MinP are usually more powerful then CCT when there are both positive and negative effects, and the differences in power values can be substantial. The reason is because when some *p*-values are very larger (e.g., one-sided *p*-values from the wrong sided-test) or some of them are negatively correlated (e.g., the *p*-values for studies with different effect directions but from the same one-sided test), CCT will result in a small test statistic and therefore a large *p*-value. Second, when two-sided *p*-values are used, CCT usually has higher power than others as expected since under these conditions, those small *p*-values are positively correlated no matter the effects have the same or different directions. Third, MCM and CMC usually have power values between those obtained by CCT and MinP. Fourth, MCM performs more similarly with MinP while CMC more similar to CCT. Similar patterns are also observed under other conditions (see Tables S3–S7 in supplementary materials).

### Real data application

We also applied the proposed tests to a real data application. Table 2 lists the estimated odds ratios (ORs) and the 95% confidence interval (CI) from a meta-analysis which includes 12 independent trials that examine the effect of patient rehabilitation designed for geriatric patients on functional outcome improvement, compared with usual care. An OR greater than 1 means the new treatment was better than the usual care. The data were taken from Figure 4 of Riley et al.^20^, part of the Figure 2 of Bachmann et al.^21^. The original meta-analysis was based on a random effect model as the Cochran’s test for homogeneity indicated that the fixed effect model is not appropriate. However, a goodness of fit test also showed that the random effect model does not fit the data either and the *p*-value combination method was suggested^22^. Based on the given estimated OR and CI, we can calculate the individual *p*-values from the 12 studies^22^. Denote U and L the upper and lower limits of the 95% CI, the test statistic can be approximated as $t=\mathrm{ln}\left(U\times L\right)/\sqrt{4\mathrm{ln}\left(U/L\right)/3.92}$, whose asymptotic null distribution is $N({0,1})$. The sample sizes of these 12 trials were relatively large, ranging from 108 and 1388, therefore, we can reasonably estimate their *p*-values using the asymptotic null distribution. For each study, three types of *p*-values, one-sided left, one-sided right, and two-sided under the three alternative hypotheses (${OR}_{i}<1, {OR}_{i}>1,\, {\text{and}} \;{OR}_{i}\ne 1,$ respectively), are calculated as shown in Table 2 and are used in the *p*-value combination tests.

**Table 2:** Data and *p*-values from 12 independent studies.

Study	OR	95% CI		*p*-value		
		Lower	Upper	Left-sided	Right-sided	Two-sided
1	1.11	0.51	2.39	0.6044	0.3956	0.7911
2	0.97	0.78	1.21	0.3928	0.6072	0.7857
3	1.13	0.73	1.72	0.7119	0.2881	0.5762
4	1.08	0.42	2.75	0.5638	0.4362	0.8724
5	0.88	0.39	1.95	0.3778	0.6222	0.7555
6	1.28	0.71	2.30	0.7948	0.2052	0.4103
7	1.19	0.69	2.08	0.7317	0.2683	0.5366
8	3.82	1.37	10.6	0.9949	0.0051	0.0102
9	1.06	0.63	1.79	0.5866	0.4134	0.8269
10	2.95	1.54	5.63	0.9995	0.0005	0.0011
11	2.36	1.18	4.72	0.9924	0.0076	0.0152
12	1.68	1.05	2.70	0.9844	0.0156	0.0313

Table 3 displays the results of the combination tests applied to the three types of *p*-values described above. Each of the three tests (Min P, Fisher chi-square test, and the z-test) is used to combine independent *p*-values from all left-sided, all right-sided, and all two-sided, separately (columns 2–4 of Table 3). The resulting two dependent *p*-values from combining left-sided *p*-values and right-sided *p*-values are further combined using CCT, MinP, MCM, and CMC. Their *p*-values are listed in the last four columns in Table 3. For instance, the *p*-values through using the z-test for combining independent *p*-values obtained from the individual left- and right-sided tests are 0.99984 and 0.00016, respectively. The two dependent *p*-values are then combined using the CCT, MinP, MCM, and CMC, we get 0.50, 0.00031, 0.00063, and 0.00063, respectively. Interestingly, when we combine the independent two-sided *p*-values, the *p*-values are 0.013, 0.0068, and 0.075 from the MinP, Fisher Chi-square, and z-test, respectively. All of them are greater than the *p*-values obtained by the MinP, MCM, and CMC tests combining two dependent *p*-values, while the CCT test has a large *p*-value of 0.5. This result indicates that appropriately combining two dependent *p*-values, each obtained through combining independent *p*-values from the same direction, is preferred to combining independent two-sided *p*-values.

**Table 3:** Results from the tests applied to a real data application.

Method combining independent *p*-values	Combined *p*-values			Method combining two dependent *p*-values			
	Left-sided	Right-sided	Two-sided	CCT	MinP	MCM	CMC
Min P	0.997	0.0064	0.013	0.99	0.013	0.026	0.22
Fisher	0.998	0.000083	0.0068	0.00017	0.00017	0.00033	0.00017
z-test	0.9998	0.00016	0.075	0.50	0.00031	0.00063	0.00063

## Discussion and conclusion

We have shown that when the significance level is small the recently proposed *p*-value combination test CCT can control type I error rate for *p*-values under arbitrary dependency structures. However, we also showed that under some conditions, CCT may be less powerful or even powerless at all. This could happen, for instance, in a genetic study, a genetic risk factor could be protective for some subpopulations, which will result in some small *p*-values and also some large *p*-values to be combined. On the other hand, the commonly used test MinP can also control type I error rate under all conditions and may be more or less powerful than CCT under some conditions. To improve the detection power, we proposed two new tests, MCM and CMC. Through a comprehensive simulation study and real data application, we showed that MCM and CMC can control type I error rate and are more robust than CCT and MinP. The proposed tests, MCM and CMC, take advantage of the two methods, CCT and MinP and therefore will maintain reasonable power under all situations. They can be applied when the dependency structures of *p*-values to be combined are unknown.

As theorem 1 shows, CCT (and also MinP, MCM, and CMC) can not obtained a *p*-value smaller than the smallest one of the *p*-values to be combined. This result suggests that when we combine independent *p*-values, we should consider other more powerful tests, such as the Fisher chi-square test, z-test and others^23^. Approaches for combining *p*-values have been extensively used in statistical practice and have significant effects on data analysis^7,10–14,24–30^. However, this research area remains challenging. Novel powerful and robust tests for combining independent and/or dependent *p*-values are still highly desired.

## Supplementary Information

Supplementary Tables.

## Data Availability

All data are presented in the paper and no additional data are available.

## Code availability

The code used to produce the results presented in this paper is available at https://github.com/zchen2020/Robust-P-value-combination-tests.
