# Supplementary material for: Robust tests for combining p-values under arbitrary dependency structures
Source: Sci Rep. 2022 Feb 24;12:3158. doi: 10.1038/s41598-022-07094-7 (PMC8873210; doi:10.1038/s41598-022-07094-7)
Supplement: Supplementary file 1 — Supplementary Tables. [file 41598_2022_7094_MOESM1_ESM.pdf]

Supplementary Tables

Table S1. Empirical type I error rate (/significance level) using 20 p-values and 10^6 replicates.

Model	Para	Test	Significance level			
			0.05	0.01	0.001	0.0001
Expo (ρ)	0.2	CCT	1.05	1.02	1.02	0.92
		MinP	0.96	0.99	1.00	0.91
		MCM	0.54	0.52	0.51	0.54
		CMC	0.99	1.00	1.01	0.92
	0.4	CCT	1.13	1.06	0.98	0.84
		MinP	0.93	0.97	0.96	0.84
		MCM	0.57	0.53	0.47	0.40
		CMC	1.00	1.01	0.97	0.84
	0.8	CCT	1.27	1.19	1.08	0.99
		MinP	0.63	0.72	0.79	0.81
		MCM	0.62	0.57	0.54	0.50
		CMC	0.93	0.94	0.93	0.93
	0.99	CCT	1.05	1.05	1.02	1.03
		MinP	0.14	0.15	0.17	0.20
		MCM	0.52	0.52	0.48	0.51
		CMC	0.58	0.59	0.57	0.57
Poly (r)	0.5	CCT	1.41	1.28	1.15	1.18
		MinP	0.77	0.88	0.99	1.13
		MCM	0.68	0.61	0.57	0.60
		CMC	1.06	1.06	1.07	1.15
	1	CCT	1.31	1.17	1.05	0.80
		MinP	0.83	0.89	0.94	0.78
		MCM	0.63	0.55	0.50	0.45
		CMC	1.04	1.03	1.00	0.79
	2	CCT	1.17	1.11	1.02	1.08
		MinP	0.87	0.94	0.94	1.02
		MCM	0.58	0.55	0.50	0.48
		CMC	1.00	1.02	0.98	1.05
	2.5	CCT	1.14	1.10	1.02	1.08
		MinP	0.86	0.94	0.94	1.04
		MCM	0.57	0.54	0.50	0.52
		CMC	0.98	1.01	0.98	1.06
SiG (d)	0.1	CCT	0.77	0.85	0.93	1.00
		MinP	0.28	0.28	0.28	0.33
		MCM	0.45	0.47	0.46	0.47
		CMC	0.50	0.56	0.59	0.59
	0.3	CCT	0.81	0.89	0.97	0.95

		MinP	0.28	0.29	0.32	0.28
		MCM	0.46	0.48	0.50	0.45
		CMC	0.53	0.58	0.65	0.66
	0.7	CCT	0.93	0.95	1.01	1.01
		MinP	0.29	0.30	0.29	0.36
		MCM	0.49	0.49	0.53	0.52
		CMC	0.58	0.61	0.65	0.65
	0.9	CCT	0.96	0.95	0.95	1.01
		MinP	0.30	0.31	0.31	0.37
		MCM	0.51	0.49	0.53	0.53
		CMC	0.60	0.61	0.64	0.62

Table S2. Empirical type I error rate (/significance level) using 100 p-values and 1e^{6} replicates.

Model	Para	Test	Significance level			
			0.05	0.01	0.001	0.0001
Expo (ρ)	0.2	CCT	1.02	1.01	1.02	0.99
		MinP	0.97	0.99	1.02	0.99
		MCM	0.54	0.52	0.50	0.49
		CMC	0.98	1.00	1.02	0.99
	0.4	CCT	1.07	1.04	1.02	0.85
		MinP	0.95	0.99	1.00	0.86
		MCM	0.55	0.53	0.49	0.47
		CMC	0.99	1.01	1.01	0.85
	0.8	CCT	1.26	1.15	1.04	1.09
		MinP	0.70	0.77	0.82	0.92
		MCM	0.61	0.57	0.51	0.51
		CMC	0.94	0.95	0.94	1.00
	0.99	CCT	1.17	1.14	1.09	1.08
		MinP	0.10	0.11	0.13	0.15
		MCM	0.58	0.56	0.55	0.59
		CMC	0.60	0.61	0.61	0.62
Poly (r)	0.5	CCT	1.63	1.36	1.09	0.92
		MinP	0.79	0.88	0.92	0.84
		MCM	0.76	0.62	0.53	0.45
		CMC	1.16	1.10	1.00	0.86
	1	CCT	1.33	1.15	1.05	1.03
		MinP	0.88	0.94	0.97	1.00
		MCM	0.63	0.56	0.53	0.48
		CMC	1.06	1.03	1.01	1.02
	2	CCT	1.13	1.07	1.05	0.92
		MinP	0.91	0.95	0.98	0.90
		MCM	0.57	0.54	0.55	0.52

		CMC	0.99	1.01	1.02	0.92
	2.5	CCT	1.11	1.07	1.02	1.09
		MinP	0.91	0.97	0.98	1.04
		MCM	0.56	0.54	0.53	0.54
		CMC	0.98	1.01	0.99	1.07
SiG (d)	0.1	CCT	0.89	0.95	0.95	1.02
		MinP	0.23	0.22	0.21	0.19
		MCM	0.48	0.48	0.46	0.57
		CMC	0.44	0.58	0.57	0.63
	0.3	CCT	0.92	0.98	1.07	1.05
		MinP	0.23	0.23	0.23	0.23
		MCM	0.49	0.50	0.55	0.50
		CMC	0.46	0.60	0.65	0.62
	0.7	CCT	1.03	1.02	0.98	0.92
		MinP	0.23	0.23	0.21	0.15
		MCM	0.52	0.51	0.46	0.49
		CMC	0.52	0.62	0.57	0.58
	0.9	CCT	1.08	1.05	1.03	1.10
		MinP	0.23	0.24	0.23	0.25
		MCM	0.54	0.53	0.53	0.53
		CMC	0.56	0.64	0.65	0.69

Table S3. Empirical power when there are 2 out of 10 p-values are significant with $\mu = 2$ and $\alpha = 0.05$.

P-value type	Test	Model (para)					
		Expo(ρ=0.5)		Poly(r=1.5)		SiG(d=0.5)	
		Different direction	Same direction	Different direction	Same direction	Different direction	Same direction
left-sided	CCT	0.47	**0.96**	0.47	**0.88**	0.37	**1.00**
	MinP	**0.75**	0.87	**0.65**	0.76	**0.60**	0.78
	MCM	0.64	0.89	0.56	0.78	0.54	0.90
	CMC	0.51	0.93	0.50	0.84	0.39	0.98
right-sided	CCT	0.47	0.00	0.46	0.00	0.45	0.00
	MinP	**0.76**	0.00	**0.65**	0.00	**0.52**	0.00
	MCM	0.65	0.00	0.55	0.00	0.48	0.00
	CMC	0.51	0.00	0.49	0.00	0.46	0.00
two-sided	CCT	**0.99**	**0.88**	**0.99**	**0.78**	**0.94**	**0.90**
	MinP	0.92	0.79	0.89	0.66	0.58	0.62
	MCM	0.95	0.79	0.93	0.67	0.72	0.69
	CMC	0.97	0.85	0.97	0.73	0.77	0.79

Table S4. Empirical power when there are 2 out of 10 p-values are significant with $\mu = 3$ and $\alpha = 0.001$.

P-value type	Test	Model (para)					
		Expo(ρ=0.5)		Poly(r=1.5)		SiG(d=0.5)	
		Different direction	Same direction	Different direction	Same direction	Different direction	Same direction
left-sided	CCT	0.50	0.99	0.50	**0.96**	0.44	1.00
	MinP	**0.92**	0.97	**0.84**	0.91	**0.87**	0.99
	MCM	0.87	0.98	0.77	0.92	0.80	1.00
	CMC	0.54	0.99	0.53	0.94	0.50	1.00
right-sided	CCT	0.50	0.00	0.49	0.00	0.52	0.00
	MinP	**0.92**	0.00	**0.84**	0.00	**0.75**	0.00
	MCM	0.87	0.00	0.77	0.00	0.69	0.00
	CMC	0.54	0.00	0.53	0.00	0.56	0.00
two-sided	CCT	1.00	0.98	1.00	0.90	1.00	1.00
	MinP	1.00	0.95	1.00	0.87	0.89	0.93
	MCM	1.00	0.95	1.00	0.87	1.00	1.00
	CMC	1.00	0.98	1.00	0.90	1.00	1.00

Table S5. Empirical power when there are 10 out of 100 p-values are significant with model= "Expo", ρ=0.5, $\mu = 2$ and $\alpha = 0.05$.

P-value type	Test	Number of significant alternatives with the same direction					
		5	6	7	8	9	10
left-sided	CCT	0.50	0.61	0.71	0.82	0.91	1.00
	MinP	**0.98**	**0.99**	**0.99**	0.99	0.99	1.00
	MCM	0.94	0.96	0.98	0.99	0.99	1.00
	CMC	0.56	0.66	0.76	0.85	0.92	1.00
right-sided	CCT	0.48	0.37	0.27	0.16	0.08	0.00
	MinP	**0.98**	**0.96**	**0.92**	**0.83**	**0.64**	0.00
	MCM	0.93	0.89	0.83	0.71	0.51	0.00
	CMC	0.54	0.43	0.33	0.21	0.11	0.00
two-sided	CCT	1.00	0.99	0.99	1.00	0.99	0.99
	MinP	0.99	0.99	0.99	0.99	0.99	0.99
	MCM	1.00	0.99	1.00	1.00	0.99	0.99
	CMC	1.00	0.99	0.99	1.00	0.99	0.99

Table S6. Empirical power when there are 10 out of 100 p-values are significant with model= "Poly", r=1.5, $\mu = 2$ and $\alpha = 0.05$.

P-value type	Test	Number of significant alternatives with the same direction					
		5	6	7	8	9	10
left-sided	CCT	0.49	0.60	0.70	0.80	0.89	1.00
	MinP	**0.96**	**0.98**	**0.99**	**0.99**	**0.99**	1.00
	MCM	0.91	0.94	0.97	0.98	0.98	1.00
	CMC	0.54	0.64	0.74	0.83	0.91	1.00

right-sided	CCT	0.49	0.38	0.28	0.18	0.09	0.00
	MinP	**0.97**	**0.95**	**0.90**	**0.82**	**0.64**	0.00
	MCM	0.92	0.88	0.81	0.70	0.50	0.00
	CMC	0.55	0.45	0.34	0.23	0.12	0.00
two-sided	CCT	0.99	1.00	1.00	1.00	0.99	0.99
	MinP	0.99	0.99	0.99	0.99	0.99	0.98
	MCM	1.00	1.00	1.00	0.99	0.99	0.99
	CMC	0.99	1.00	1.00	1.00	0.99	0.99

Table S7. Empirical power when there are 10 out of 100 p-values are significant with model= "SiG", d=0.5, $\mu = 2$ and $\alpha = 0.05$.

P-value type	Test	Number of significant alternatives with the same direction					
		5	6	7	8	9	10
left-sided	CCT	0.48	0.58	0.67	0.77	0.87	**1.00**
	MinP	**0.74**	**0.80**	**0.84**	0.88	0.91	0.93
	MCM	0.69	0.76	0.83	**0.89**	**0.94**	0.99
	CMC	0.52	0.61	0.71	0.80	0.89	0.99
right-sided	CCT	0.45	0.35	0.27	0.18	0.10	0.00
	MinP	**0.70**	**0.62**	**0.52**	**0.41**	**0.27**	0.00
	MCM	0.64	0.55	0.45	0.33	0.20	0.00
	CMC	0.48	0.39	0.30	0.20	0.11	0.00
two-sided	CCT	**0.99**	**0.99**	**1.00**	**0.99**	**0.99**	**0.99**
	MinP	0.79	0.80	0.80	0.82	0.82	0.83
	MCM	0.96	0.97	0.97	0.97	0.97	0.94
	CMC	0.98	0.98	0.98	0.98	0.98	0.97
